# Recent advances in graves ophthalmopathy medical therapy: a comprehensive literature review

**DOI:** 10.1007/s10792-022-02537-6

**Published:** 2022-10-22

**Authors:** Xueting Li, Senmao Li, Wanlin Fan, Alexander C. Rokohl, Sitong Ju, Xiaojun Ju, Yongwei Guo, Ludwig M. Heindl

**Affiliations:** 1 Department of Ophthalmology, Faculty of Medicine and University Hospital Cologne, University of Cologne, Kerpener Strasse 62, 50937 Cologne, Germany; 2 Eye Center, Second Affiliated Hospital, Zhejiang University School of Medicine, Jiefang Road 88, Hangzhou, Zhejiang, 310009 China; 3 Center for Integrated Oncology (CIO), Aachen-Bonn-Cologne-Duesseldorf, Cologne, Germany

**Keywords:** Graves ophthalmopathy, Thyroid-associated ophthalmopathy, Diagnosis, Management

## Abstract

Graves ophthalmopathy (GO), which occurs in autoimmune thyroid disease, can reduce patients’ quality of life due to its impact on visual function, physical appearance, and emotional health. Corticosteroids have been the first-line treatment for GO. More recently, the pathogenesis of GO has made significant progress. Various targeting biological agents and immunosuppressive agents make GO management more promising. Fully understanding GO pathogenesis and precise clinical management are beneficial for the prognosis of patients. Therefore, we conducted a comprehensive review of the medical management of GO and summarized research developments to highlight future research issues.

## Introduction

Graves ophthalmopathy (GO) is also known as thyroid-associated ophthalmopathy (TAO), thyroid eye disease (TED), or Graves orbitopathy. GO is mostly a thyroid-related organ autoimmune disease. It mainly occurs in patients with hyperthyroidsm and can also be accompanied by euthyroidism, subclinical or overt hypothyroidism [1]. The prevalence rate of GO in Europe is around 90–155/100,000 [2]. The proportion of Graves’ patients with all grades of GO, particularly those with severe disease, appears to decline over time [3]. Women are more prone to the GO than men. All ages could suffer from GO, but the incidence peaks between 30 and 50 years old [4]. The histopathologic changes such as immune cell infiltration, hyaluronan deposition, and lipogenesis result in orbital tissue expansion and muscle hypertrophy. The characteristic manifestations, including eyelid contracture, proptosis, and even sight loss, burden society and families and seriously endanger people’s health and quality of life [5]. This review provides a concise overview of GO, focusing on the causes and recent advances in drug therapy.

## Methods

We conducted structured searches on GO in PubMed and Web of Science. The search strategy included keywords, i.e., Graves ophthalmopathy, thyroid-associated ophthalmopathy, thyroid eye disease, Graves orbitopathy, diagnosis, and treatment. The related references until July 2022 are also traced. Since some diagnoses were published in the 1960s, the past 40 years’ studies are all included. Most of the literature cited for GO medical management has been published in recent five years. Only English literature has been included.

## Pathogenesis

### Immune pathway

**Fig. 1:**
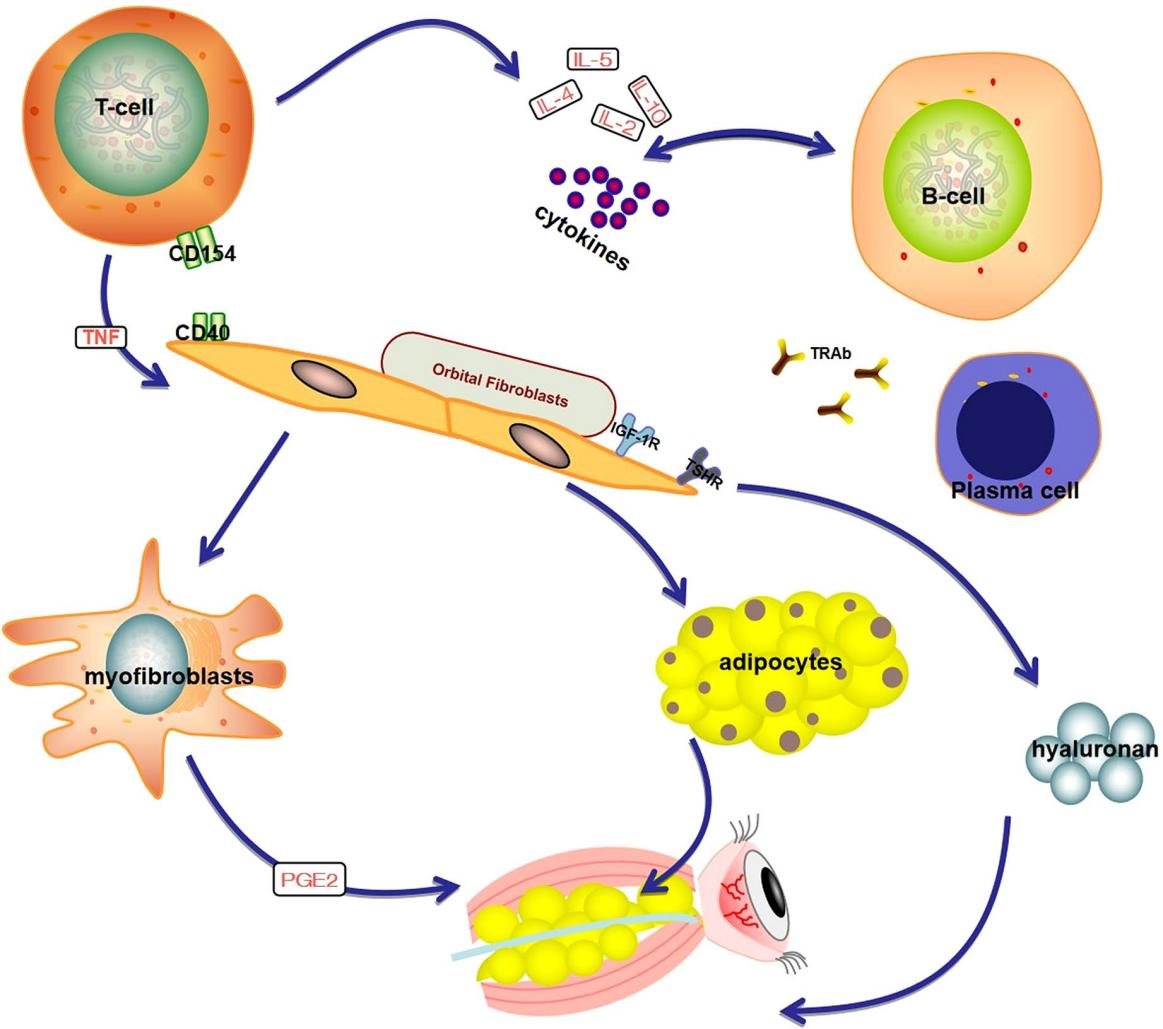
Graves Ophthalmopathy pathogenesis. Autoreactive T cells interact with orbital fibroblasts (OFs) via CD40:CD154 ligation. B cells differentiate into plasma cells, which synthesize and secrete TRAb. TRAb can mediate the autoimmune response of GO by recognizing TSHR. Activated OFs then secrete various inflammatory cytokines. TSHR and IGF-1R are expressed in the common area of the OFs membrane and exist in the form of a complex which plays a synergistic role in the functional regulation of orbital fibroblasts. Activated OFs can differentiate into either myofibroblasts or adipocytes and promote hyaluronan synthesis. As a result, orbital soft tissue expansion and congestion in GO. *Created with BioRender.com*

GO can be divided into progressive and stable phases, including orbital tissue inflammation, expansion, remodeling, and fibrosis in succession. The GO pathogenesis is shown in Fig. 1. The progressive phase involves inflammation and infiltration of orbital tissue by Type 1 T helper (Th 1) cells, B-lymphocytes, mast cells, and macrophages. B‑cells migrate to the orbit and are activated by recognizing TSHR or IGF‑1R and bound by the T‑cells. B‑cells are further activated by interleukin (IL) 4 and differentiate into plasma cells that produce autoantibodies. The infiltrated immune cells interact with the orbital fibroblasts (OFs) either via cellular interactions, e.g., CD40-CD154 ligation, or via various cytokines and growth factors, e.g., cytokines, chemokines, and stimulatory autoantibodies (GD-IgG) [6]. This leads to the activation of OFs, which express relatively high levels of functional thyrotropin receptor (TSHR) and insulin-like growth factor 1 receptor (IGF-1R) [7]. OFs can be divided into two main subgroups according to the expression of cell surface marker Thy-1 (CD90): CD90 + OFs, which are prone to differentiate into myofibroblasts, and CD90‑ OFs, which are mostly transformed into adipocytes [6]. When stimulated by different types of cytokines, the balance of CD90‑ OFs and CD90 + OFs determines whether the pathological changes are adipogenesis or fibrosis, leading to orbital fat expansion or fibrosis, and the symptoms, signs, and disease course of patients will also be different [8]. The extracellular matrix synthesis (especially hyaluronan) also contributes to tissue expansion and remodeling [6].

### Oxidative stress

Reactive oxygen species (ROS) are produced during cell metabolism, including superoxide and hydrogen peroxide (H_2_O_2_). The over-formed ROS will be eliminated through enzymatic and non-enzymatic mechanisms during transferring one electron from the redox donor to molecular oxygen (O_2_) [9]. Oxidative stress occurs when the balance between ROS production and elimination is disrupted, resulting in peroxide and free radicals’ accumulation, subsequent causing remarkable damage to proteins, lipids, membranes, and nucleic acids in cells, and ultimately leading to mitochondrial dysfunction and a loss of enzymatic activity [10].

GO is in a status of high oxidative stress, which promotes the occurrence and development of diseases through its effect on orbital fibroblasts. Oxidative stress can enhance the patients’ inflammatory response by promoting the proliferation of orbital fibroblasts and the expression of inflammatory mediators [11]. The inflammatory response will further aggravate oxidative stress and keep periorbital tissues in a state of inflammation. The oxidative stress response of GO will accelerate the exhaustion of the enzymatic antioxidant and further reduce the ability to resist oxidative stress. In addition, the thyroid hormone overproduction of GD also leads to a hypermetabolic state with increased oxygen and intracellular adenosine triphosphate (ATP) consumption and ROS generation, which in turn damages thyrocytes that exacerbate autoimmune reactions as well as extrathyroidal tissues [12, 13].

## Risk factors

The risk factors of GO can be divided into endogenous (unmodifiable) and exogenous (modifiable). Endogenous factors include age, race, gender, and genetics. Exogenous factors include thyroid dysfunction, smoking, radioactive iodine (RAI), hypercholesterolemia, oxidative stress, and diabetes mellitus (DM). The multigenic conditions, gene–gene, and gene-environment interactions should be considered to affect the development of GO [14].

### Endogenous factors

GO is known to be a multifactorial and polygenic disease. Currently, thyroid-specific genes (e.g., the TSH-R gene) and sensitivity genes, e.g., human leucocyte antigen (HLA) and cytotoxic T-lymphocyte antigen-4 (CTLA-4), are closely related to the occurrence and progression of GO. Lahooti et al. also found that the CASQ1 gene polymorphism is related to GO, and the CASQ1 gene expression is significantly up-regulated in the thyroid tissue of GO patients [15]. Several genes were discovered to be differentially expressed in OFs from GO patients, but the relative importance of each gene has yet to be determined [16].

### Exogenous factors

#### Thyroid dysfunction

Both hyper and hypothyroidism can influence the severity of GO [17, 18]. Euthyroidism should be promptly restored and stably maintained in all GO patients [19]. Thyrotropin receptor antibody (TRAb) level is an independent risk factor of GO. TRAb is divided into three groups according to its interaction with the TSH-R [20], and current evidence indicates that thyroid-stimulating antibodies (TSAb) are more closely associated with GO [21, 22]. TSAb enters the bloodstream and induces differentiation of OFs and secretion of hydrophilic glycosaminoglycans (GAG), leading to edema and later fibrosis [23]. Accordingly, great attention should be paid to ocular changes in GD patients with high titers of TSAb. TRAb is significantly higher in GO patients than in non-GO patients. Eckstein et al. followed 159 GO patients for 12–24 months and described that the TRAb trigger and constantly maintain the autoimmune process in orbit [24]. The continuous measurement of TRAb could prevent more severe clinical outcomes.

#### Smoking

Smoking is considered to be the most significant exogenous risk factor for GO. Hägg and Asplund et al. firstly proposed a direct relationship between smoking and severe GO in 1987 [25]. In addition, the amount of tobacco, even past smoking, is related to the severity of the disease in GO patients [26]. Compared to nonsmokers, active smokers suffer from a higher degree of GO severity and higher rates of poor response to therapies [27, 28]. Smoking might induce tissue remodeling through hypoxia, oxygen-free radical generation, and stimulation of adipogenesis [29].

#### Radioiodine treatment

RAI is used alone or with other medications or surgery to treat GO. However, hypothyroidism may occur after radiation exposure by autoimmune thyroiditis and thyroid atrophy [30]. RAI was verified to be associated with progression or worsening of GO [31], but these are often transient [32] and can be prevented with prophylactic glucocorticoids [33]. The 2021 EUGOGO guidelines recommended 0.3–0.5 mg/kg/body weight as starting dose, tapered, and withdrawn after three months for RAI-treated patients at high risk of progression or de novo development; 0.1–0.2 mg/kg/bodyweight, tapered and withdrawn after six weeks for low-risk patients [19]. Steroid cover is unnecessary for the inactive GO if hypothyroidism is avoided and other GO progression risk factors, particularly smoking and high serum TSHR-Ab titers, are absent.

#### Hypercholesterolemia

Recent studies showed that the CAS was higher in patients with high cholesterol, suggesting a role of cholesterol itself in the development of GO [34–36]. Hypercholesterolemia is related to oxidative stress and the release of proinflammatory cytokines, resulting in systemic proinflammatory actions [35]. In addition, hypercholesterolemia can also lead to tissue inflammation by activating Toll-Like Receptor 4-Myeloid Differentiation Factor 2 (TLR4–MD2) [37].

## Clinical manifestation and diagnosis

General clinical manifestation of GO includes exophthalmos, lid retractions, diplopia, and decreased visual acuity. In severe conditions, pain, cornea exposure, dysthyroid optic neuropathy (DON), and vision loss can also be found [38]. DON may also result in characteristic changes in color vision, contrast sensitivity, pupillary examination, and automated visual field perimetry [39]. For GO diagnosis, visual acuity, slit lamp, ultrasound, CT, and MRI scan can be helpful. In addition, the high concentration of proteins in tear fluid makes tear sampling and testing an important non-invasive technique to investigate the mechanism and diagnose GO [40]. Tear levels of some proteins were significantly elevated in GD patients with moderate/ severe GO compared to GD without GO [41, 42]. Numerous studies have also proposed potential protein biomarkers for GO, allowing the early diagnosis and progress prediction of the GO [43, 44]. After diagnosing, physicians should assess the clinical disease activity by Clinical Activity Score (CAS) [19, 45] and severity by NOSPECS [46, 47], VISA [19, 48], and European Group on Graves Orbitopathy (EUGOGO) classification [49, 50] (Table 1).

**Table 1:** GO assessment criteria

Criteria	Category	Pros and cons
Clinical activity score	Spontaneous retrobulbar pain; 2. Pain on attempted upward or downward gaze; 3. Redness of eyelids; 4. Redness of conjunctiva;5. Swelling of caruncle or plica; 6. Swelling of eyelids; 7. Swelling of conjunctiva (chemosis). A ten-item CAS, including an increase in exophthalmos ≥ 2 mm, a decrease of eye movements in any direction of gaze ≥ 8˚, and a decrease of visual acuity ≥ 1line on the Snellen chart during a period of 1–3 months	A binary scale (yes/no). Each clinical feature was given equal weight, and the scoring standard of "yes" or "no" can only be judged when the symptom appears or relieves. A ten-item CAS is better to evaluate recent progression
NOSPECS	0 No physical signs or symptoms 1 Only signs, no symptoms (signs limited to upper lid retraction, stare, and lid lag) 2 Soft-tissue involvement (symptoms and signs) 3 Proptosis 4 Extraocular muscle involvement 5 Corneal involvements 6 Sight loss (optic nerve involvement)	The first systematic classification method for the eye changes of GO. The progression of GO is not always in regular succession according to the classes
VISA classification	Vision (visual blurring, color desaturation); Inflammation (orbital aching at rest or with movement, eyelid or conjunctival swelling or redness); Strabismus (diplopia, with horizontal or vertical gaze, intermittent in primary gaze, constant in primary gaze); Appearance changes (distress about bulging eyes, eyelid retraction, and/ or fat pockets, dry eye symptoms)	The definition of severity in appearance is unclear. An adequate validation is not available
EUGOGO	Mild GO: GO have a minor impact on daily life. One or more of the following: Minor lid retraction (< 2 mm); mild soft-tissue involvement; exophthalmos; < 3 mm above normal for race and gender; no or intermittent diplopia, and corneal exposure responsive to lubricants; Moderate-to-severe GO: GO has sufficient impact on daily life to justify the risks of immunosuppression (if active) or surgical intervention (if inactive). Two or more of the following: lid retraction ≥ 2 mm; moderate or severe soft-tissue involvement; exophthalmos ≥ 3 mm above normal for race and gender; inconstant or constant diplopia; Sight-threatening GO: Patients with dysthyroid optic neuropathy (DON) and/ or corneal breakdown	The EUGOGO classification has been validated both in clinical and research studies

## Management

The management of GO depends on the patients’ symptoms and severity, consisting of fundamental, medical, and surgical treatments. The ideal treatment goal is to decrease the risk of visual complications, minimize side effects, eliminate the demand for surgical interventions, restore thyroid function and avoid progression or recurrence of GO.

The early diagnosis and preventive management, i.e., removal of modifiable risk factors, may reduce the prevalence and change the clinical manifestations of GO. Generally, fundamental treatments are recommended regardless of the severity, i.e., artificial tears (ointment are needed when corneal exposure occurs), dark glasses, parasol, raising the pillow at night, and controlling risk factors [51]. Most mild GO patients have no progressive development of the affected extraocular muscles, and a wait-and-see strategy is feasible. A minority of mild GO patients may be suggested for low-dose immunomodulation if GO is active. Moderate-to-severe and active GO patients with obvious symptoms and active disease are mainly treated with medical or orbital irradiation therapy. For DON, it is necessary to be treated with a high dose of IVMP immediately. If patients are non-responsive or intolerant within two weeks, urgent orbital decompression is recommended to be performed. If DON is improved or resolved after two weeks, weekly IVMP should be continued to manage moderate-to-severe and active GO.

In 20–30% of GO patients, rehabilitative surgeries are needed to correct severe signs [52]. Elective rehabilitative surgery is suitable for improving the visual function and QoL after GO has been inactive for at least six months. When more than one surgical procedure is required, orbital decompression surgery, eye muscle, and eyelid surgery should be performed in the given order.

### Nonspecific therapies

#### Antioxidants

Selenium (Se), a common antioxidant, can decompose excessively generated ROS and play a protective role against large-scale oxidative stress. Se is crucial in the antioxidant defense system and maintains a stable redox state. Se could prevent necrosis and apoptosis by counteracting the proliferation of orbital fibroblasts induced by H_2_O_2_ and inhibiting the release of proinflammatory cytokines and hyaluronic acid [53]. Se supplementation is shown to reduce the impact of high oxidative stress levels in GO patients by reducing the level of TRAb, thyroglobulin antibody (TGAb), and thyroid peroxidase antibody (TPOAb) [54]. A multicenter, double-blind clinical trial proved that the course of GO could be improved after six months’ continuous Se supplementation (100mcg twice a day), and the treatment effects were still maintained after six months [55]. However, the patients included are all in the selenium-deficient area. Se status was rarely evaluated in the clinic before the Se supplementation [56]. The action of selenium for the long-term outcome and whether the same beneficial effects can be found in regions without deficient selenium remains to be well cleared [57].

In addition to Se, many antioxidants are available in nature, such as quercetin, vitamin C, N-acetyl-l-cysteine, melatonin, and β-carotene, and exert functions in reducing the level of oxidative stress. Quercetin has anti-proliferative properties and can reduce orbital fibroblasts proliferation via necrosis and hyaluronic acid (HA) release. However, quercetin may sustain and worsen the autoimmune reaction because further exposure to endogenous antigens worsens the prognosis [58]. Vitamin C and N-acetyl-l-cysteine reduced cell proliferation in GO. N-Acetyl-l-cysteine can also reduce the release of HA, IFNγ, and IL1β, but TNFα is unaffected [59]. Melatonin may not be a strong candidate, considering it cannot affect fibroblast proliferation, but it lacks toxicity and affects fibroblast functions, i.e., HA and cytokines release. It may incorporate other antioxidants in a mixture to be administered to GO patients [59]. β-carotene, displaying antioxidant, anti-inflammatory, and antiproliferation effects, seems promising [60] but needs to be further analyzed in clinical practice.

#### Corticosteroids

Corticosteroids have been the mainstay treatment since the 1950s but are often less effective and associated with side effects, e.g., cushingoid features, weight gain, palpitation, myalgia, hypertension, liver damage, and cardiovascular and cerebrovascular events [61, 62]. Ocular steroid injection avoids systemic complications but can cause local complications, e.g., globe perforation, toxic ON, lower eyelid herniation of orbital fat, or arterial occlusion [63]. IVMP has the advantages of better efficacy and lower incidence of adverse reactions than OGC. The side effects of oral treatment can be minimized by IVMP [64]. Stiebel-Kalish and coworkers evaluated 1367 patients from 33 trials and concluded that IVMP has a small but statistically significant advantage in treatment effects and causes fewer adverse events over oral glucocorticoids (OGC) [65]. Gao et al. also reported that intravenous glucocorticoid pulse (IVGC) was markedly more effective than OGC, and IVGC + orbital radiation was more effective than OGC + orbital radiation [66].

As already recommended in the 2021EUGOGO guideline [19], the combination of a moderate cumulative dose of IVMP (0.5 g/week/6 weeks) and a moderate daily dose of mycophenolate sodium(0.72 g/day/6 weeks)is recommended as the first line of treatment. Alternative treatment is a high single dose of IVMP (0.75 g/week/6 weeks). If the response is poor or absent, second-line treatments can be planned, i.e., the second course of GCs, orbital radiotherapy with oral or intravenous glucocorticoids, the combination of azathioprine, or cyclosporine with oral prednisone, teprotumumab, tocilizumab, or rituximab (RTX). In addition, monthly monitoring during the treatment is necessary. Patients should also be screened for recent hepatitis, liver function, severe hypertension, inadequately managed diabetes, cardiovascular morbidity, and glaucoma before IVMP.

#### Mycophenolate mofetil

Mycophenolic acid (MPA), a selective immunosuppressant, inhibits inosine monophosphate dehydrogenase, blocks DNA synthesis, and has cytostatic effects on B and T cells [67]. MPA can inhibit the differentiation and generation of T lymphocytes and the synthesis of immune markers on the surface of immune cells. If the cell metabolism and proliferation are active, the inhibitory effect of MPA will be vital. Hence, it has a strong target for the treatment of GO. Ye and collaborators evaluated and compared the efficacy and safety of GCs to MMF in 174 patients with active moderate-to-severe GO. Patients are treated with IVMP at 0.5 g per day for three consecutive days a week for two weeks, then oral prednisone at 60 mg daily for eight weeks, followed by a gradual reduction plan of 5 mg per week for 14 weeks. The mycophenolate mofetil (MMF) group found a more significant overall response rate (91.3% vs. 67.9%, p < 0.001), a better CAS response (92.5% vs. 70.5% improvement, *p* < 0.05), and a significant improvement rate of diplopia and proptosis (90.4% and 68.8% improvement, respectively), indicating that MMF has a particular effect on active GO [68]. The add-on mycophenolate benefit was also detected in ophthalmic symptoms and signs at 24 weeks and 36 weeks by post-hoc analysis in patients with active and moderate-to-severe GO in a multicenter, observer-masked trial [69]. However, its widespread use in clinics is restricted because the cost of MMF oral capsules is relatively high (an oral capsule of 250 mg is around $51). In addition, MMF cause dose-dependent gastrointestinal (GI) side effects, including ulcers, diarrhea, and nausea [70]. Enteric-coated mycophenolate sodium (EC-MPS), a delayed-release formulation, improves GI tolerability [71, 72].

#### Cyclosporine

Cyclosporine can inhibit the activation of cytotoxic T lymphocytes. It also can reduce the antigen expression of monocytes and macrophages, activating inhibitory T lymphocytes and further inhibiting the production of cytokines. Furthermore, cyclosporine has a specific synergistic effect combined with oral GCs, reducing hormone resistance, and continuing its anti-inflammatory effects after the cessation of glucocorticoids. Concerning ocular outcome and recurrence rate, cyclosporine combined with GCs can achieve a better curative rate than cyclosporine alone [73]. Since the adverse effects of cyclosporine (dose-dependent liver and renal toxicities, lung damage, and hypertension, among others) cannot be overlooked [74], cyclosporine is generally recommended to be administered in combination with oral GCs rather than itself alone.

#### Azathioprine

Azathioprine, an anti-proliferative medicine, has been used as a steroid-sparing therapy. A post hoc analysis illustrates that azathioprine might improve 48-week clinical outcomes in combination with glucocorticoids to treat active moderate-to-severe GO [75]. It suggests that azathioprine may potentially prevent the long-term recurrence of GO after steroid discontinuation. Azathioprine-related adverse effects, including bone marrow suppression, nausea, and vomiting, have been extensively studied because of its widely used in many other autoimmune diseases [76, 77].

#### Statins

The effects of statins could be attributed to their ability to decrease cholesterol, pleiotropic anti-inflammatory actions, interact with methylprednisolone, and influence tissue remodeling [35, 78]. The use of statins is considered to increase the effectiveness of the immunosuppressive medication, decreasing the dosage of GC and thereby reducing their side effects. The results of both basic and clinical studies on the impact of statins on GO are promising [79–81]. A large register-based study examined the effect of statin therapy on the incidence of GO among 34,894 newly diagnosed GD and reported that statins, instead of other lipid-lowering agents, may protect against the development of GO [81]. A recent phase 2 randomized clinical trial suggested that the combination of atorvastatin and ivGCs regimen improves the outcome of moderate-to-severe active GO [80]. However, a phase 3 clinical trial with a longer follow-up is still needed before introducing statins into the clinic.

### Targeted molecular therapies

#### Tumor Necrosis Factor α Inhibitors

GO pathogenesis is related to the up-regulation of proinflammatory cytokines, e.g., tumor necrosis factor-α (TNF-α). TNF-α antagonists, e.g., imatinib mesylate, adalimumab, etanercept, thalidomide, and infliximab [82], may be used as steroidal protective drugs to treat GO. Ten mild-to-moderate GO patients were subcutaneously injected with etanercept 25 mg twice weekly. After three months of treatment, the mean CAS decreased from 4 to 1.6 (60%). However, three patients developed recurrence of GO after discontinuation of the etanercept [83]. Van Steensel et al. studied the potential efficacy of imatinib mesylate and adalimumab through a whole orbital tissue culture system [84]. The results showed that imatinib mesylate reduces inflammation and tissue remodeling. Adalimumab can reduce IL-6 production but is ineffective enough to affect orbital hyaluronan production. A case series showed a reduced inflammatory response in 6/12 patients [85]. No improvement of proptosis or motility was reported. Further RCTs are necessary to determine the efficacy of TNF-α inhibitors in GO management.

#### Rituximab

RTX is a monoclonal antibody against the human CD20 antigen. RTX inhibits B-cell activation and mediates specific decreases in thyroid-peroxidase antibody and IgM levels [86, 87]. RTX is a relatively safe and feasible treatment method in moderate to severe GO. It can significantly reduce the CAS after treatment, especially after previous corticosteroid failure [49]. The minor adverse effects, including fever, throat itching, infusion reaction, and nausea, can be relieved by slowing down RTX infusion or intravenous hydrocortisone. Several studies have investigated the efficacy of RTX in GO management. The outcomes are not entirely consistent. Stan et al. concluded that RTX in the placebo-controlled trial is not efficacious [88]. Salvi et al. found RTX quite effective in patients with moderate to severe GO in better ocular mobility, visual function, and decreased surgery demand [89]. Li et al. also reported the promising prospect of RTX combined with orbital radiation. The noticeable baseline inconsistency related to differences in subjects’ age, gender, and smoking history may contribute to the discrepancy. According to a study by Eid et al., RTX only offered limited and partial improvement in active moderate-to-severe GO patients with a long duration of disease. Response to RTX therapy appears to be significantly influenced by GO duration. Even though it is not advised as a first-line treatment, RTX appears to have the potential to control active moderate-to-severe GO, especially when applied in the early active stage of the disease [90]. RTX can be used as one of several options for patients who are not sensitive to or do not respond to IVMP.

#### Tocilizumab

Tocilizumab, a recombinant humanized monoclonal anti-IL-6 receptor inhibitor, is used to treat inflammatory and autoimmune conditions. It was first described in the literature as a humanized anti-interleukin (IL)-6 receptor monoclonal antibody MRA Chugai in 2003 [91]. The first study on tocilizumab was a prospective interventional nonrandomized study with 18 steroid-resistant GO patients in 2014. Improvement occurred after intravenous tocilizumab treatment with a mean CAS score reduction of 5.89 ± 1.41 points, and the mean TSI (thyroid-stimulating immunoglobulin) levels were significantly lower. All adverse events reported were mild, with mostly minor events such as tiredness, neutropenia, upper respiratory tract infection, and slight elevation of liver enzymes [92]. The first RCT with tocilizumab, in 2018, double-masked, showed 93.3% of tocilizumab-treated patients improved at least 2 points by CAS, and greater improvement in EUGOGO score and a more considerable reduction of exophthalmos compared to placebo. One patient experienced a moderately raised transaminase, and another experienced acute pyelonephritis [93]. Tocilizumab can also be injected subcutaneously, which improves patient compliance and convenience [94]. More large sample size clinical trials are needed to verify the efficacy of tocilizumab in active, moderate-to-severe GO patients.

#### Teprotumumab

Teprotumumab, an insulin-like growth factor-1 receptor inhibitor, reduces the TSH-stimulated proinflammatory cytokines secretion of isolated fibrocytes [95] and significantly decreases the expression of TSH-R and IGF-1R in fibrocytes [96]. Teprotumumab significantly improved ptosis, diplopia, CAS score, and quality of life in moderate-to-severe GO and showed a favorable safety profile [97, 98]. A randomized, double-blind, placebo-controlled trial was conducted in 22 centers worldwide in 2017. The results showed that teprotumumab could improve proptosis and reduce CAS in moderately to severely active GO patients with only 5% complications incidence [97]. A placebo-controlled teprotumumab phase III clinical trial showed exophthalmos has decreased by ≥ 2 mm in 82.9% moderately to severely active GO at the 24^th^ week [98]. Due to disease-modifying properties, teprotumumab was approved “breakthrough therapy” designation by the U.S. Food and Drug Administration (FDA) in 2020, which marks a significant milestone for GO management. Although the novel drug teprotumumab is promising, we still don’t entirely understand its indications and limitations [99]. The problems related to the high price [100], the durability of the favorable response [101], and the safety of teprotumumab should be solved. Complete recording and reporting of the actual incidence of the adverse event, including muscle spasms, nausea [98], hyperglycemia, and hearing impairment [102, 103], are warranted. Accordingly, it is not recommended to expand the therapeutic indications without rigorous clinical trial evidence demonstrating the efficacy and safety [104].

## Conclusions

Early intervention of GO may alter the disease course and significantly improve long-term outcomes. Only by fully understanding the complex cytokine network of GO pathogenesis and cross-integrating with immunology and pharmacology can we break through the bottleneck and benefit patients. Researchers have made headway in GO mechanisms and immunosuppressants in recent years, bringing new hope for GO management. The clinical research process of GO medicine is different, and further studies are merited for their safety and efficacy.

## Data Availability

Not applicable.
